# Genomic Architecture of the Two Cold-Adapted Genera *Exiguobacterium* and *Psychrobacter*: Evidence of Functional Reduction in the *Exiguobacterium antarcticum* B7 Genome

**DOI:** 10.1093/gbe/evy029

**Published:** 2018-02-08

**Authors:** Larissa M Dias, Adriana R C Folador, Amanda M Oliveira, Rommel T J Ramos, Artur Silva, Rafael A Baraúna

**Affiliations:** Laboratory of Genomics and Bioinformatics, Center of Genomics and Systems Biology, Institute of Biological Sciences, Federal University of Pará, Belém, PA, Brazil

**Keywords:** *Psychrobacter*, *Exiguobacterium*, cold adaptation, psychrotrophic, comparative genomics, extremophiles

## Abstract

*Exiguobacterium* and *Psychrobacter* are bacterial genera with several cold-adapted species. These extremophiles are commonly isolated from the same habitats in Earth’s cryosphere and have great ecological and biotechnological relevance. Thus, through comparative genomic analyses, it was possible to understand the functional diversity of these psychrotrophic and psychrophilic species and present new insights into the microbial adaptation to cold. The nucleotide identity between *Exiguobacterium* genomes was >90%. Three genomic islands were identified in the *E. antarcticum* B7 genome. These islands contained genes involved in flagella biosynthesis and chemotaxis, as well as enzymes for carotenoid biosynthesis. Clustering of cold shock proteins by Ka/Ks ratio suggests the occurrence of a positive selection over these genes. Neighbor-joining clustering of complete genomes showed that the *E. sibiricum* was the most closely related to *E. antarcticum*. A total of 92 genes were shared between *Exiguobacterium* and *Psychrobacter*. A reduction in the genomic content of *E. antarcticum* B7 was observed. It presented the smallest genome size of its genus and a lower number of genes because of the loss of many gene families compared with the other genomes. In our study, eight genomes of *Exiguobacterium* and *Psychrobacter* were compared and analysed. *Psychrobacter* showed higher genomic plasticity and *E. antarcticum* B7 presented a large decrease in genomic content without changing its ability to grow in cold environments.

## Introduction

Several cold-adapted bacterial strains are taxonomically classified in the genera *Exiguobacterium* and *Psychrobacter* (Vishnivetskaya et al. 2000; Rodrigues et al. 2006; Carneiro et al. 2012) and are commonly described in environmental studies using microbiological or molecular approaches (Rodrigues et al. 2006, 2009; Yadav et al. 2015). Strains of these genera commonly colonize the same ecological niche, expressing genes that produce a cold-adaptive phenotype. *Psychrobacter* species have been isolated from the deep water of the sea, permafrosts, Antarctic glacial ice, and sediment, among other habitats (Bowman et al. 1997; Shivaji et al. 2005; Chaturvedi and Shivaji 2006; Moyer and Morita 2007; Shravage et al. 2007; Rodrigues et al. 2009). The genus *Psychrobacter* was first described by Juni and Heym (1986), whereas *Exiguobacterium* was first described by Collins et al. (1983) with the microbiological characterization of the species *E. aurantiacum*.

The genus *Exiguobacterium* is physiologically more diverse than *Psychrobacter*, and it includes psychrotrophic, mesophilic, and moderate thermophilic bacterial species (Vishnivetskaya and Kathariou 2005). Strains of *Exiguobacterium* are commonly isolated from glacial ice, hot springs, the rhizosphere of plants, permafrosts, and tropical and temperate soils (Rodrigues et al. 2006; Rodrigues and Tiedje 2007; Carneiro et al. 2012). *Exiguobacterium* and *Psychrobacter* are phylogenetically distant genera. Whereas *Exiguobacterium* is classified in the phylum Firmicutes, class *Bacilli*, and order *Bacillales*, *Psychrobacter* is classified in the phylum Proteobacteria, class *Gammaproteobacteria*, order *Pseudomonadales*, family *Moraxellaceae* (https://www.namesforlife.com/; last accessed January 20, 2017). Despite their taxonomical classification, both genera have developed molecular mechanisms when they are grown in harsh conditions, such as low temperature environments.

The mechanisms of adaptation include the following: 1) increased enzymatic catalytic efficiency; 2) production of unsaturated branched-chain fatty acids to maintain membrane fluidity; 3) expression of cold shock proteins that stabilize the bacterial cytosol at low temperatures; 4) uptake of compatible solutes to maintain the cellular osmotic balance; and 5) carotenoid production (Barria et al. 2013; De Maayer et al. 2014). Because of these metabolic peculiarities, psychrophilic and psychrotrophic bacteria have great biotechnological appeal and are widely used in studies of biodegradation of hydrocarbons, antibiotics, and other environmental pollutants (Jiang et al. 2014; Bajaj and Slingh 2015). Furthermore, these micro-organisms are of extreme importance to better understand the ecological and biogeochemical processes of the Earth’s cryosphere (Boetius et al. 2015).

With the advent of Next-Generation Sequencing (NGS) technologies in the last decade, the number of genomes available in databases has increased. In total, the GenBank database has 42 genomes of *Exiguobacterium*, four of which are completely assembled and belong to psychrotrophic or psychrophilic species. *Psychrobacter* contains 37 deposited genomes, four of which are completely assembled and belong to cold-adapted species. Several bioinformatics tools have been developed to compare and analyse this large amount of genomic data. Our work presents the results of a comparative genomic analysis performed with the genera *Exiguobacterium* and *Psychrobacter*. The results obtained helped us understand and visualize the adaptive molecular diversity of these two phylogenetically distant but ecologically similar cold-adapted genera.

## Materials and Methods

### Data Collection

We have carefully reviewed the 79 genomes of both genera deposited in GenBank and selected four genomes for each genus to perform the comparative analysis. The selection was based on two criteria: 1) the “habitat” and “growth temperature” information contained in the literature or BioSample data and 2) the completeness of the genomes. Only psychrotrophic or psychrophilic species with genomes that were completely assembled were selected for analysis. The genome of *Exiguobacterium antarcticum* B7 was sequenced by our research group using a hybrid assembly methodology that used fragments and mate-paired libraries (Carneiro et al. 2012). This genome was deposited under the accession number CP003063.1. The remaining seven genomes were obtained from NCBI’s GenBank database and have the following accession numbers: CP001022.1 (*Exiguobacterium sibiricum* 255-15), CP015731.1 (*Exiguobacterium* sp. U13-1), CP006866.1 (*Exiguobacterium* sp. MH3), CP000082.1 (*Psychrobacter arcticus* 273-4), CP014945.1 (*Psychrobacter alimentarius* PAMC 27889), CP012678.1 (*Psychrobacter urativorans* R10.10B), and CP000323.1 (*Psychrobacter cryohalolentis* K5).

### General Genomic Comparisons

Initially, all genomes were compared with the genome of *E. antarcticum* B7 by BLASTn, generating a ring on the software BRIG v.0.95 (Alikhan et al. 2011). The GenBank file of *E. antarcticum* B7 was manually curated to highlight the main genes involved in the cold adaptation processes. These genes were indicated in the rings generated by the software, BRIG. Synteny between the genomes was analysed on the software Artemis Comparison Tool (ACT) v.13.0.0 (Carver et al. 2005). Genomic Islands (GIs) of *E. antarcticum* B7 were predicted by GIPSy (Soares et al. 2016). Four types of islands were predicted: Resistance Island (RI), Pathogenicity Island (PAI), Symbiotic Island (SI), and Metabolic Island (MI). The thermophilic specie *Exiguobacterium* AT1b was used as reference. Genes within GIs were compared by BLASTn to the database of Predicted Genome Islands (Pre_GI) using an e-value of 1e-05 to determine the main taxonomic hosts of the foreign genes. Pre_GI contains a sequence database of genes acquired by horizontal transfer for the Bacteria and Archaea domains (Pierneef et al. 2015).

To determine the conservation of cold shock proteins (CSPs), all sequences were extracted from GenBank files and a database was created using the script gb2fasta.py of the BlastGraph v.1.0 program package (Ye et al. 2013). This database was compared with itself by BLASTp using the package Blastall (Altschul et al. 1990). The resulting .xml file containing the similarity scores was analysed in BlastGraph software to construct a de Brujin graph where each node represents a protein sequence and the size of the edges represents the degree of similarity between these sequences (Ye et al. 2013). A neighbor-joining tree was calculated in MEGA7 (Kumar et al. 2016) using the same data set described above for BlastGraph analyses. Sequences were aligned using ClustalW before tree calculation. A total of 1,000 replicates were calculated. To evaluate the selective pressure on CSP genes, the sequences were analysed using the web application Ka/Ks of the Norwegian Bioinformatics Platform (Siltberg and Liberles 2002).

### Clustering Analysis

To evaluate the functional and nucleotide similarity between strains of *Exiguobacterium* and *Psychrobacter* two approaches were performed, both of which were based on clustering methods. In the first approach, a clustering based on the neighbor-joining model was performed to compare the DNA sequence of the genes from the core genome. The core genome and the distance matrix were calculated by PGAP v.1.11 (Zhao et al. 2012) using a coverage cutoff of 80% and identity of 50%. The output file 4.PanBased.NJ.tree was analysed in the software SplitsTree v.4.14.2 (Huson and Bryant 2006) to obtain an unrooted tree. In the second approach, the whole nucleotide sequence of the genomes, including their plasmids, were compared all-against-all by BLASTn using the software Gegenees v.2.2.1 (Agren et al. 2012) with a fragment size of 200 bp and a step size of 100 bp. Fragments with identity values >30% were used to calculate the similarity scores. The result was presented as a heat map with nucleotide similarity in percentage. The heat map was subsequently analysed in the software SplitsTree v.4.14.2 to obtain an unrooted tree using the neighbor-joining model.

### Gene Distribution

The gene distribution was analysed with the software PGAP v.1.11 using the same parameters described in the section above. First, the pangenome calculation was performed separately for each genus to extract the information about the core genome, accessory genes, singletons, and paralogous genes. Subsequently, the pangenome was calculated using all genomes from both genera. In the latter analyses, the PGAP output file, 1.Orthologs_Cluster.txt, and the PGAP input file .pep containing the peptide sequence of all genomes was used to extract the amino acid sequence of the core genes by using a Perl script developed by our research group called getFastaFromOrthologs.pl. The core genes were classified into Gene Ontology (GO) categories using the software Blast2GO (Conesa et al. 2005). Venn diagrams and bar graphs were obtained in the R package.

### Gene Gain and Loss Analysis

The level of gain and loss of the gene families was analysed in the software BlastGraph v.1.0 (Ye et al. 2013). Initially, a multifasta file containing all of the protein sequences from the *Exiguobacterium* and *Psychrobacter* genomes was created using a script provided by the software package. This multifasta file was compared against itself by BLASTp using the blastall package. The distance matrix in .xml format was used as an input file for the software BlastGraph. A phylogenetic tree was calculated using the UPGMA (Unweighted Pair Group Method with Arithmetic Mean) model and a bootstrap of 1,000 replicates. To determine the main biological subsystems of the genomes, their complete sequence was submitted as a fasta file to the Rapid Annotation using Subsystem Technology (RAST) server (Aziz et al. 2008). Subsystem classification was evaluated, and the main pathways involved in cold adaptation were compared using the RAST server.

## Results and Discussion

### Comparative and Clustering Analysis

**Fig. 1.**
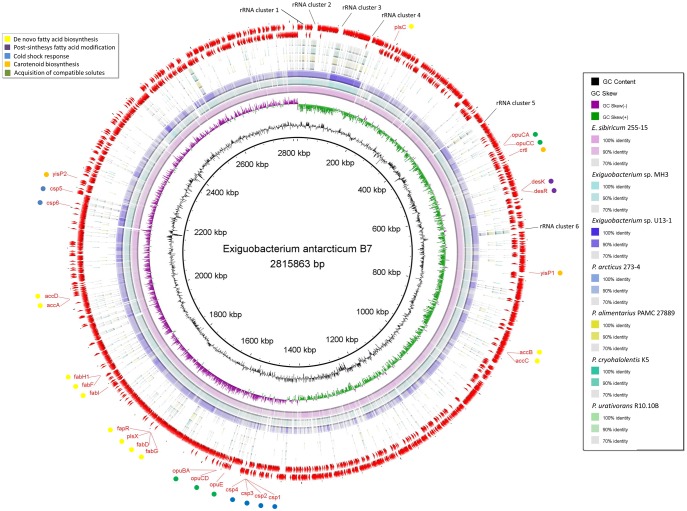
—**Circular map designed to compare the nucleotide identity of all genomes against *Exiguobacterium antarcticum* B7.** The genomes were compared by BLASTn, and the percent identity between them was determined by the intensity of color in the circular rings. The innermost ring to the outermost in this figure is presented as follows: the GC content and CG skew of *E. antarcticum* B7, the genomes of *E. sibiricum* 255-15, *Exiguobacterium* sp. MH3, *Exiguobacterium* sp. U13-1, *Psychrobacter arcticus* 273-4, *P. alimentarius* PAMC 27889, *P. cryohalolentis* K5, and *P. urativorans* R10.10B, respectively. The three outermost rings comprise the location of the GIs (yellow arcs) and CDSs (red arcs) of *E. antarcticum* B7. The main genes involved in cold adaptation are indicated by circles colored according to the metabolic pathway.

**Fig. 2.**
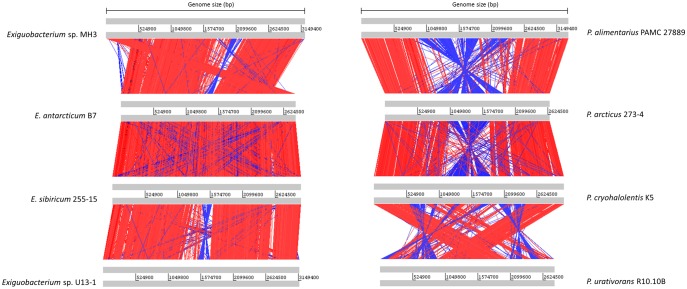
—**Analysis of genomic synteny.** Synteny plots were obtained using the Artemis Comparison Tool. Gray lines indicate the genome size of each bacterial strain. Red bars indicate the conserved genomic regions, and blue bars indicate regions of genomic inversion. To better visualize the structural correlation between genomes, a minimum cut-off of 150 for BLASTn scores was applied. *Psychrobacter* strains have a significant number of inversions in their genomes. *Exiguobacterium* strains present a more conserved structural correlation.

The eight selected genomes varied considerably in size, number of predicted genes, GC content, presence of plasmids, and other genotypic characteristics (table 1). As revealed by the rings of figure 1, a high nucleotide identity (between 90% and 100%) within *Exiguobacterium* species was observed. Compared with the *Psychrobacter* genomes, only seven regions of *E. antarcticum* B7 showed an identity near 100%. Using the genome browser, it was possible to note that these conserved regions carried the rRNA gene clusters (fig. 1). A small number of genomic inversions were observed between the genomes of *Exiguobacterium* strains. On the other hand, *Psychrobacter* genomes showed a larger number of inversions and larger inverted regions (fig. 2). *Exiguobacterium antarcticum* B7 e *E. sibiricum* 255-15 are the species with the highest structural similarity. Although *E. antarcticum* B7 has the smallest genome of the genus, no large insertion/deletion regions could be observed in the synteny graph (fig. 2).

**Table 1:** General Characteristics of the Bacterial Species

Organism	Isolation Source	Minimum Growth Temperature	Genome Size (bp)	Number of CDSs	GC Content (%)	Reference
*Exiguobacterium antarcticum* B7	Ginger Lake, Antarctica	−2°C	2,815,863	2,736	47.50	Carneiro et al. (2012)
*E. sibiricum* 255-15	Permafrost, Siberia	−10°C	3,034,136	3,107	47.68	Rodrigues et al. (2008)
*Exiguobacterium* sp. MH3	Rhizosphere of a duckweed strain, *Lemna minor*	4°C	3,164,195	3,160	47.20	Tang et al. (2013)
*Exiguobacterium* sp. U13-1	Lake Untersee, Antarctica	–^a^	3,208,634	3,178	47.10	Fomenkov et al. (2017)
*Psychrobacter arcticus* 273-4	Kolyma, Siberia	−10°C	2,650,701	2,130	42.8	Ayala-del-Rio et al. (2010)
*P. cryohalolentis* K5	Kolyma Lowland, Russia	−10°C	3,059,876	2,510	42.3	Unpublished data
*P.urativorans* R10.10B	Soil	20°C	2,802,354	2,359	42.2	Unpublished data
*P. alimentarius* PAMC 27889	Rocky desert, Antarctica	−10°C	3,332,539	2,678	42.9	Lee et al. (2016)

^a^ Data not available in BioSample or literature.

GIPSy was used to predict genomic islands in *E. antarcticum* B7. The prediction was based on commonly genomic features such as GC content; codon usage; presence of transposase genes; virulence, metabolism, antibiotic resistance, or symbiosis factors; flanking tRNA genes; and absence of the predicted islands in closely related species (Soares et al. 2016). Two PAIs, three RIs, and one SI were detected (supplementary table S1, Supplementary Material online). These Genomic Islands (GIs) were tagged with the acronyms EaPAI (*E. antarcticum* Pathogenicity Island), EaRI, and EaSI, respectively. We did not found any evidence of horizontal gene flow in the genomic region of the islands using the Pre_GI database. Interestingly, EaPAI_1 was predicted in the same location as EaRI_2, as well as EaPAI_2 was predicted in the same location as EaRI_3 and Ea_SI_1 (supplementary table S1, Supplementary Material online). EaPAI_1 and EaRI_2 (genomic position: 2,229,960 up to 2,257,989 bp) contain genes involved in flagella biosynthesis and chemotaxis, as well as genes encoding enzymes involved in the early stages of carotenoid biosynthesis. A two-component system regulated by a histidine kinase was also described within the island. In EaPAI_2, EaRI_3 and EaSI_1 (genomic position: 2,459,471 up to 2,469,289 bp), five of the ten CDSs detected were uncharacterized proteins. The other five CDSs were identified by computational homology as UDP-N-acetylglucosamine 2-epimerase (*wecB*), Uracil phosphoribosyltransferase (*upp*), Serine hydroxymethyltransferase (*glyA*) and, once again, a two-component system (composed of a histidine kinase and a regulatory protein). The gene *wecB* encodes an enzyme that catalyzes the synthesis of a bacterial capsule precursor which could explain the prediction of this region as a putative pathogenicity island.

One of the main mechanisms of cold adaptation in species of the order Bacillales is the expression of the DesR-DesK two-component system. During cold stress, a membrane sensor histidine kinase (DesK) activates a regulatory protein (DesR) that in turn positively regulates the expression of fatty acid desaturase genes (*des*) (Aguilar et al. 2001). Fatty acid desaturase enzymes modify the chemical structure of membrane fatty acids in order to maintain membrane fluidity.

*Exiguobacterium antarcticum* B7 contains twelve two-component systems throughout its genome. However, none of them were near a fatty acid desaturase gene as described for *Bacillus subtilis* (Aguilar et al. 2001). Additionally, all two-component systems identified showed low similarity with the model system of *B. subtilis* identified by Aguilar et al. (2001) (BLASTp identity values were <50%). Two *des* genes were identified in the genome of *E. antarcticum* and were upregulated during cold stress (Dall’Agnol et al. 2014) suggesting that desaturase enzymes are under regulatory control and as observed in *B. subtillis*, regulate chemical composition of membrane fatty acids.

**Fig. 3.**
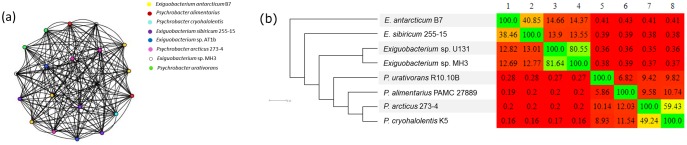
—**De Bruijn graph of cold-shock proteins and clustering analysis of genomes.** (*a*) A de Brujin graph clustering the sequences of CSPs (nodes) according to the results of the reciprocal BLASTp (edges). The comparison was conducted with the blastall package. The graph was designed in the BlastGraph program. (*b*) All-against-all comparison of the nucleotide genome sequences. The heat map represents the percent identity between the genomes. The tree was calculated in Gegenees software using the neighbor-joining model.

**Fig. 4.**
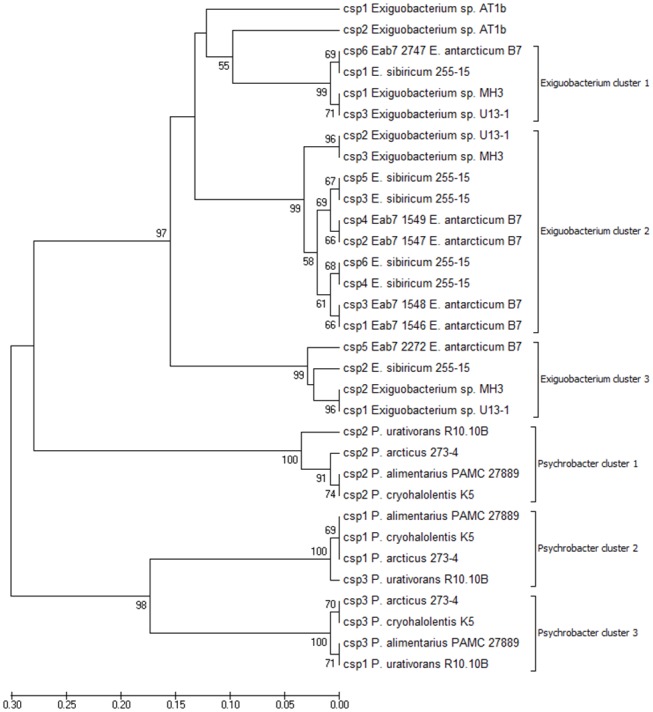
—**Phenetic clustering of CSP sequences using the UPGMA method.** The optimal tree with the sum of branch length = 1.74505027 is shown. The percentage of replicate trees in which the associated taxa clustered together in the bootstrap test (1,000 replicates) are shown next to the branches. Analyses was conducted in MEGA7.

The extracted CSP sequences were compared all-against-all using the blastall package. A de Brujin graph was obtained using BlastGraph (fig. 3*a*) to visualize the sequences similarity based on the reciprocal BLASTp values. Nodes of figure 3*a* represent the protein sequences, and the size of the edges represents the degree of similarity (reciprocal BLASTp) between these sequences. No significant differences were observed among CSPs from *Exiguobacterium* and *Psychrobacter*. The CSPs of the thermophilic bacteria *Exiguobacterium* sp. AT1b also clustered together with all other proteins (fig. 3*a*). Additional analysis was performed using the phenetic clustering method UPGMA. In this analysis, CSPs were clustered into two groups according to the bacterial genera (fig. 4). In addition, an intracluster analysis demonstrated that CSPs from both genera could be divided on three different clades supported by high bootstrap values (fig. 4). It is worth noting that *E. antarcticum* B7 and *E. sibiricum* 255 have six CSP genes each, while the other species have only three. This gene duplication could be an important mechanism of adaptation to cold environments. However, two of these CSPs of *E. antarcticum* B7 (locus_tag: EaB7_2272 and EaB7_2747) were downregulated after 72 h of growth at 0°C (Dall’Agnol et al. 2014) suggesting that these proteins are not necessary for cold acclimation. Therefore, they possibly play different roles from those observed for the other CSPs.

The clustering by *K*_a_/*K*_s_ ratio (nonsynonymous and synonymous substitution rates) was used to evaluate the selective pressure on these CSP genes. We noted that only CSPs that were downregulated in cold temperatures showed very low Ka/Ks ratio (0.1255 and 0.3256) suggesting a purifying selection to conserve their protein sequence and function (supplementary fig. S1 and table S2, Supplementary Material online). Nonsynonimous substitutions were more prevalent (Ka/Ks ratio >1) in the nodes 4 and 5 of the tree (supplementary fig. S1 and table S2, Supplementary Material online). These high values of Ka/Ks ratio clustered CSPs into three groups suggesting that these groups are evolving at different rates. The differential expression of CSP genes in cold temperatures is an indicative of distinct roles of these proteins. It is worth noting that the gene clusters observed in UPGMA analysis are different from what was observed in the Ka/Ks ratio analysis (fig. 4 and supplementary fig. S1, Supplementary Material online).

For phylogenomic comparisons a dendogram was calculated using the heat map of similarity generated by Gegenees software (fig. 3*b*). In the dendrogram, *P. cryohalolentis* and *P. arcticus* are the most closely related species sharing 49–59% of nucleotide similarity (fig. 3*b*). *Exiguobacterium antarcticum* B7 and *E. sibiricum* 255-15 shared 38–40% of nucleotide similarity (fig. 3*b*). The other *Exiguobacterium* genomes showed higher identity values (80.55–81.64%), although they have been isolated from different ecological niches (table 1). Strains U131 and MH3 showed low identity with *E. antarcticum* (12.82% and 12.69%, respectively) and *E. sibiricum* (13.01% and 12.77%) despite their classification in the same genus. In addition, a high value of split weight was observed dividing the two genera (3,919.75), thus evidencing the high phenetic distance between these taxa (supplementary fig. S2, Supplementary Material online). The values of split weight are drastically reduced within each genus clade. The branch length represented as a split weight shows the depth of the divergence between the taxa (supplementary fig. S2, Supplementary Material online).

### Gene Distribution

**Fig. 5.**
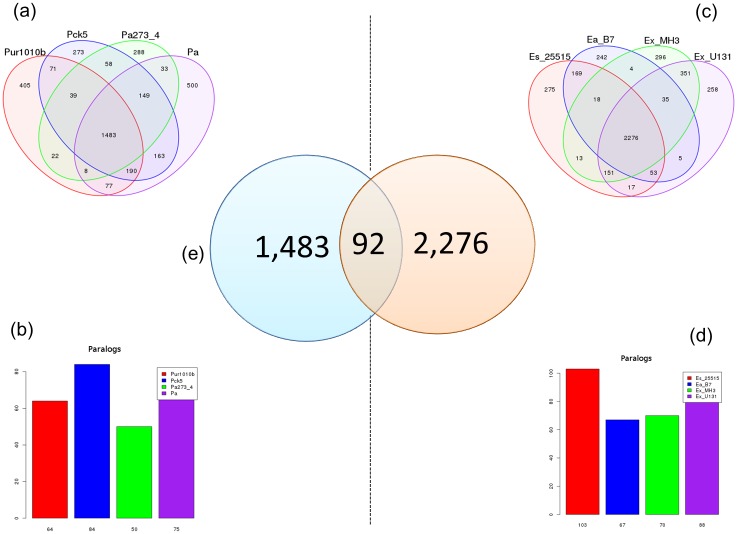
—**Venn diagram and bar plots showing the results of the gene distribution calculated in PGAP.** (*a*) Venn diagram with the number of genes shared among *Psychrobacter urativorans* R10.10B (red), *P. cryohalolentis* K5 (blue), *P. arcticus* 273-4 (green), and *P. alimentarius* PAMC 27889 (purple). (*b*) Bar plot showing the number of paralogous genes in each strain of *Psychrobacter*. (*c*) Venn diagram with the number of genes shared among *Exiguobacterium sibiricum* 255-15 (red), *E. antarcticum* B7 (blue), *Exiguobacterium* sp. MH3 (green), and *Exiguobacterium* sp. U13-1 (purple). (*d*) Bar plot showing the number of paralogous genes in each strain of *Exiguobacterium*. (*e*) Shared and singleton genes between the species of *Exiguobacterium* and *Psychrobacter*.

**Fig. 6.**
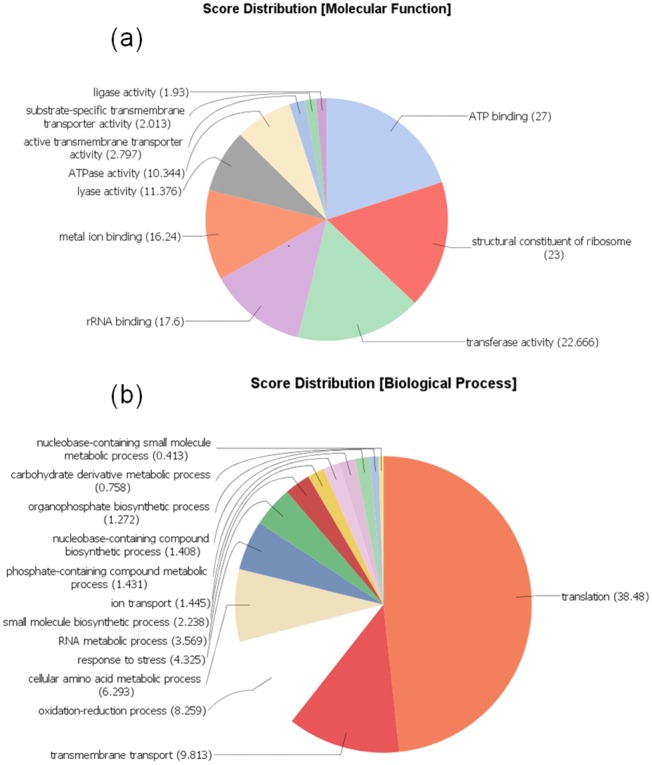
—**Molecular Functions and Biological Processes of gene ontology for the 93 genes shared among *Exiguobacterium* and *Psychrobacter* species.** Analysis was performed with Blast2GO software. (*a*) Level 3 of the molecular functions. (*b*) Level 3 of the biological processes.

In our study, 2,276 genes were shared among the four species of *Exiguobacterium* (fig. 5*c*), and 1,483 genes were shared among the four species of *Psychrobacter* (fig. 5*a*). It was observed that only 92 genes were shared between strains of *Exiguobacterium* and *Psychrobacter* (fig. 5*e*) (supplementary table S3, Supplementary Material online). One of the CSPs was identified among these core genes (EaB7_1549). Therefore, this is a highly conserved mechanism present in phylogenetically distant taxa. The core genes were subsequently classified into GO terms (fig. 6). CSPs were classified into the “response to stress” group of the GO Biological Processes (fig. 6*b*). They have notorious importance to cold adaptation by maintaining cell viability through the stabilization of the secondary structures of nucleic acids (Barria et al. 2013). Recently, several other functions of CSPs were described, such as their assistance in cellular osmotic balance, protection against oxidative stress and starvation (Keto-Timonen et al. 2016).

Four chaperone genes were also classified into the “response to stress” group of the GO Biological Processes, including an ATP-dependent chaperone ClpB and chaperone DnaK (fig. 6*b*). Studies using the model bacterium *Escherichia coli* have shown that ClpB is a translocase that acts in the absence or presence of DnaK by assisting unfolded or misfolded proteins in returning to their native structure (Li et al. 2015). Despite their importance to cytosol stabilization, Dall’Agnol et al. (2014) showed that ClpB and DnaK of *E. antarcticum* B7 are downregulated under low temperatures.

The most represented biological process among the core genes was “translation” (38 of the 93 proteins) (fig. 6*b*). The L2 and L3 50 S ribosomal proteins are examples of gene products that are involved in the translation process. The other most represented biological processes were transmembrane transport (9.8%), oxidation–reduction (8.2%), and cellular amino acid metabolism (6.2%) (fig. 6*b*). Thus, much of the shared genetic information among *Exiguobacterium* and *Psychrobacter* is composed of housekeeping genes. The main molecular functions described for the core genes were ATP binding (27%), structural constituent of ribosome (23%), transferase activity (22.6%), rRNA binding (17.6%), and metal ion binding (16.2%) (fig. 6*a*).

Many paralogous genes were identified in all the strains. *Psychrobacter cryohalolentis* K5, *P. alimentarius* PAMC 27889, *P. urativorans* R10.10B, and *P. arcticus* 273-4 showed 84, 75, 64, and 50 paralogous genes, respectively (fig. 5*b*). *Exiguobacterium sibiricum* 255-15, *Exiguobacterium* sp. U13-1, *Exiguobacterium* sp. MH3, and *E. antarcticum* B7 showed 103, 88, 70, and 67 paralogous genes, respectively (fig. 5*d*). Fatty acid desaturase proteins were found in the core genes of *Exiguobacterium* species but were absent in the shared genes of *Psychrobacter*. As previously mentioned, this enzyme is regulated by a two-component system, and is involved in the insertion of double carbon bonds in fatty acid chains linked to the cell membrane (Los and Murata 1998; Sakamoto and Murata 2002). A total of 79 hypothetical proteins were detected in *E. antarcticum* B7. Functional determination of these hypothetical proteins is one of the main bottlenecks in the postgenomic era. Bioinformatic approaches have been applied to the inference of protein function, such as protein–protein interaction networks and homology modeling (Galperin and Koonin 2004; Piovesan et al. 2015).

### Gene Gain and Loss Analysis

**Fig. 7.**
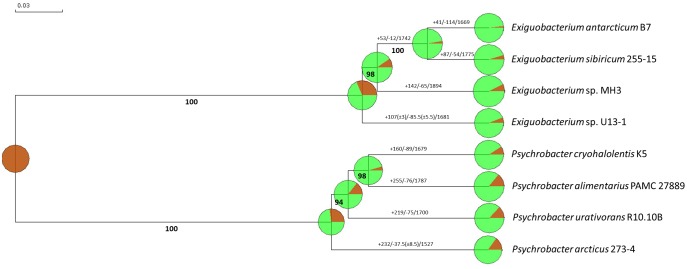
—**Evidence of gene gain and loss using reciprocal BLASTp analysis.** The dendrogram was obtained in the BlastGraph software. Bold numbers represent the percentage of bootstrap values. Numbers in each branch of the tree represent the number of gene families that were gained (+) or lost (−) compared with their closest ancestor. The circular graph is a graphical representation of the gain/loss analysis. The green color represents the conserved families while the brown color represents the gained families.

In the neighbor-joining clustering presented in figure 7, it is shown that *E. antarcticum* B7 has the lowest genetic content of its genus, followed by *E. sibiricum* 255-15, *Exiguobacterium* sp. MH3, and *Exiguobacterium* sp. U13-1. The size of the genomes is indicated by the diameter of the circular graph. Additionally, *E. antarcticum* B7 is the only species among all genomes analysed that lost more gene families than it gained (+41/−114) (fig. 7). All other species of both cold-adapted genera presented an increase in the number of gene families compared with their closest ancestor. Therefore, *E. antarcticum* B7 needs less genetic information to grow under low temperatures when compared with other species of the same genus.

The analysis of the subsystem categories performed with RAST showed a significantly higher number of genes for the *Psychrobacter* species classified in the category “Cofactors, vitamins, prosthetic groups, pigments” (table 2). The number of genes for biotin biosynthesis were notoriously greater in the *Psychrobacter* species. On the other hand, the *Exiguobacterium* species showed a significant number of genes involved in motility and chemotaxis. Genes of this subsystem were not detected in the *Psychrobacter* genomes. This observation is consistent with the lifestyle and ecological niche of each of the two bacterial genera. *Exiguobacterium* strains have peritrichous flagella being commonly isolated from aquatic ecosystems.

**Table 2:** Percentage of Genes Distributed According to the Subsystem Category for Each Genome

Subsystem Category	Number of Features							
	*Exiguobacterium****antarcticum* B7**	*E. sibiricum* 255-15	*Exiguobacterium* sp. MH3	*Exiguobacterium* sp. U13-1	*Psychrobacter cryohalolentis* K5	*P. arcticus* 273-4	*P. alimentarius* PAMC 27889	*P. urativorans* R10.10B
Cofactors, vitamins, prosthetic groups, pigments	5.1%	5.3%	5%	5%	8.9%	8.5%	8.5%	8.5%
Cell wall and capsule	3.4%	3.3%	2.8%	3.3%	4.7%	4.8%	4.1%	4.1%
Virulence, disease, and defense	1.8%	1.7%	1.9%	1.9%	2.6%	2.2%	1.9%	1.9%
Potassium metabolism	0.4%	0.4%	0.5%	0.5%	0.5%	0.6%	0.6%	0.6%
Photosynthesis	0.1%	0%	0.1%	0.09%	0%	0%	0%	0%
Miscellaneous	0.9%	0.8%	0.9%	0.9%	1.1%	0.8%	1%	1%
Phages, prophages, transposable elements, plasmids	0.06%	0.09%	0.3%	0.06%	0%	0.5%	0.5%	0.5%
Membrane transport	2.6%	0.02%	2.9%	2.9%	3.1%	2.9%	2.4%	2.4%
Iron acquisition and metabolism	0.6%	0.7%	0.9%	0.8%	0.6%	0.2%	0.2%	0.2%
RNA metabolism	4.5%	4.5%	4.3%	4.3%	6.5%	6.8%	6.7%	6.7%
Nucleosides and nucleotides	3.9%	3.7%	3.6%	3.5%	3.5%	5.2%	3.3%	3.3%
Protein metabolism	6.8%	5.8%	7.7%	7.5%	9.2%	9.5%	8.5%	8.5%
Cell division and cell cycle	1.6%	1.4%	1.4%	1.4%	1.2%	1.3%	1.3%	1.3%
Motility and chemotaxis	2.5%	2.3%	2.3%	2.3%	0%	0%	0%	0%
Regulation and cell signaling	1.4%	1.6%	1.4%	1.6%	2.2%	1.8%	2.3%	2.3%
Secondary metabolism	0.1%	0.1%	0.1%	0.1%	0.1%	0.2%	0.2%	0.2%
DNA metabolism	2.2%	2.3%	2%	2%	3.9%	3.7%	3.1%	3.1%
Fatty acids, lipids, and isoprenoids	4.1%	3.9%	3.9%	3.9%	5.3%	5.3%	4.7%	4.7%
Nitrogen metabolism	0.4%	0.5%	0.4%	0.4%	0.6%	0.9%	0.6%	0.6%
Dormancy and sporulation	0.4%	0.4%	0.4%	0.4%	0.07%	0%	0.1%	0.1%
Respiration	1.6%	1.8%	1.5%	1.5%	4.2%	4.0%	4.2%	4.2%
Stress response	2.8%	2.8%	2.6%	2.6%	3.6%	3.5%	3.4%	3.4%
Metabolism of aromatic compounds	0.1%	0.1%	0.2%	0.2%	1.0%	0.3%	0.2%	0.2%
Amino acids and derivatives	10.3%	9.8%	8.8%	8.8%	13.4%	11.1%	12.4%	12.4%
Sulfur metabolism	0.3%	0.3%	0.3%	0.3%	0.8%	0.9%	0.9%	0.9%
Phosphorous metabolism	1.4%	1.2%	1.1%	1.1%	1.1%	1.1%	1.3%	1.3%
Carbohydrates	9.2%	10.5%	10.6%	10.5%	8.1%	8.1%	6.7%	6.7%

Note.—Only the names of the strains are presented in the table. Percentage was calculated taking into consideration the total number of CDSs predicted by RAST server.

## Conclusions

Cold habitats comprise ∼20% of Earth’s surface (Fountain et al. 2012) and have been successfully colonized by species from all three domains of life. The importance of these communities ranges from their biotechnological applications (Feller 2013) to studies of astrobiology (Pikuta and Hoover 2003). In microbial ecology, several species have been isolated from the poles of our planet and many other psychrotrophic and psychrophilic species have been found in environments where cold is uncommon. In our study, we compared the genomes of eight cold-adapted species from *Exiguobacterium* and *Psychrobacter* genera.

The genetic content of these two genera were quite distinct both functionally and structurally. Nevertheless, one cold shock protein, which is considered essential for survival at low temperatures, were one of the few proteins shared between the genera. The genes coding for CSPs of *E. antarcticum* B7 were clustered into three groups that are apparently undergoing positive selective pressure. The number of genes shared among the species of *Psychrobacter* is lower than that observed for *Exiguobacterium*, indicating a greater genomic plasticity of this first genus. Interestingly, *Psychrobacter* has a more restricted ecological distribution, whereas *Exiguobacterium*, with less genomic plasticity, is commonly isolated from several types of environments (Rodrigues et al. 2009).

Additionally, the cold-adapted species sequenced by our laboratory, *E. antarcticum* B7, presented a considerable reduction in the number of gene families compared with the other species analysed, but it maintained its capacity to grow at low temperatures (Dall’Agnol et al. 2014). Other important genetic modifications are also observed, which allow the ecological adaptation of the studied species, such as an increase in the number of genes for flagella formation in *E. antarcticum* B7, which was isolated from an aqueous polar environment.

## Supplementary Material

Supplementary data are available at *Genome Biology and Evolution* online.

## Authors’ Contributions

Analysed and interpreted the data from this study and wrote the manuscript: L.M.D., A.R.C.F., and A.M.O. Developed in-house scripts and contributed to the bioinformatics analyses: R.T.J.R. Conceived the study, conducted the analysis and wrote the manuscript: A.S. and R.A.B. All authors have read and approved the final version of the manuscript.

## Supplementary Material

Supplementary Data
