# Supplementary material for: Genomic Architecture of the Two Cold-Adapted Genera Exiguobacterium and Psychrobacter: Evidence of Functional Reduction in the Exiguobacterium antarcticum B7 Genome
Source: Genome Biol Evol. 2018 Feb 8;10(3):731–41. doi: 10.1093/gbe/evy029 (PMC5833320; doi:10.1093/gbe/evy029)
Supplement: Supplementary Data [file evy029_supp.pdf]

Supplementary Material

The Genomic Architecture of the Two Cold-Adapted Genera *Exiguobacterium* and *Psychrobacter*: Evidence of Functional Reduction in *Exiguobacterium antarcticum* B7 genome

Larissa M. Dias[1†], Adriana R. C. Folador[1†], Amanda M. Oliveira[1], Rommel T.J. Ramos[1], Artur Silva[1], Rafael A. Baraúna[1*]

[1] *Laboratory of Genomics and Bioinformatics, Center of Genomics and Systems Biology, Institute of Biological Sciences, Federal University of Pará, 66075-110, Belém, PA, Brazil.*

[*] Author for correspondence: Rafael A. Baraúna, Laboratório de Genômica e Bioinformática, Centro de Genômica e Biologia de Sistemas, Rua augusto Corrêa, Nº 01, 66075-110, Guamá, Belém/PA, Brasil, Email: rabarauna@ufpa.br

Supplementary Figures.

Fig. S1. Clustering of cold shock protein sequences according to the ratio of nonsynonymous (ka) to synonymous (Ks) nucleotide substitution rates. Red lines divide groups of sequences that showed Ka/Ks ratio above 1 (selective positive pressure). Green numbers represent the nodes of table S2. Blue numbers represent the Ka and Ks values of each branch. Locus tag of each gene is shown in parentheses.

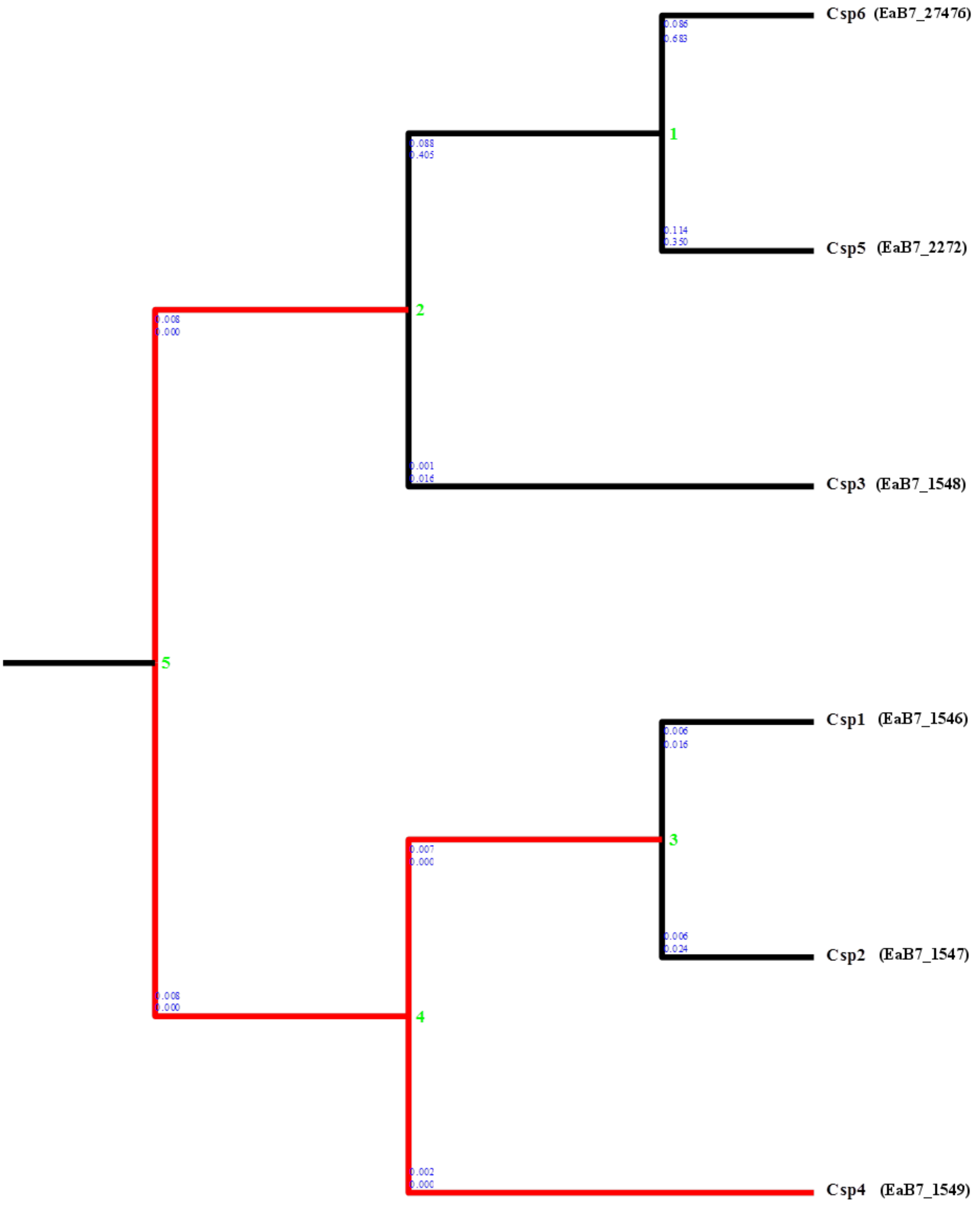

Fig. S2. Phylogenomic analysis using the core genes of *Exiguobacterium* and *Psychrobacter*. The tree was calculated in PGAP using the neighbour-joining model. The value of the split weight is presented in the branch that separates the two bacterial clades. The split weight shows the depth of the divergence between the clades of *Exiguobacterium* and *Psychrobacter*.

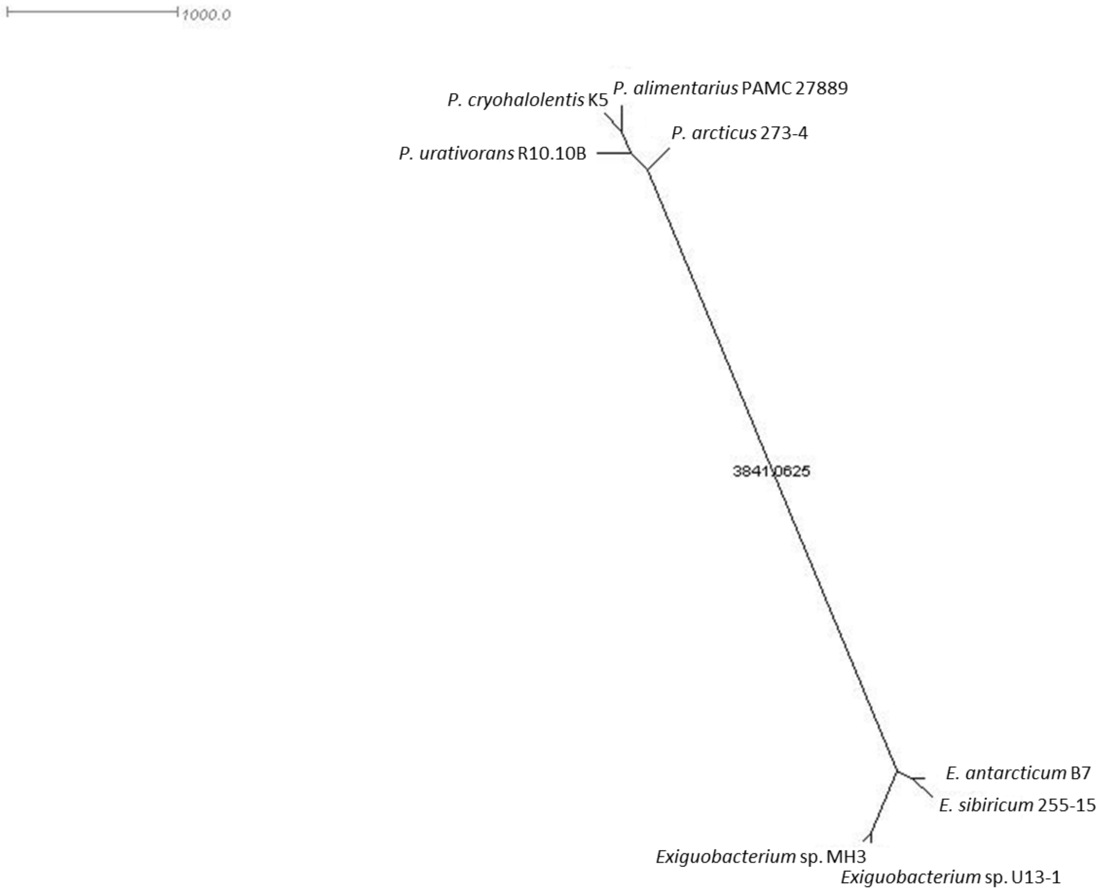

Supplementary Tables

Table S1. List of CDSs detected within GIs of *E. antarcticum* B7 genome. Pathogenicity Islands, Resistance Islands and Symbiotic Islands are abbreviated to EaPAI, EaRI and EaSI, respectively.

Genomic Island	Locus_tag	Product name	Gene name	Island Location (pb)
EaPAI_1 / EaRI_2				2229960..2257989
	Eab7_2274	Hypothetical protein	-	
	Eab7_2275[a]	Hypothetical protein	-	
	Eab7_2277	Flagellar biosynthesis, FlhS-related protein	-	
	Eab7_2278	Flagellar protein FliS	*fliS*	
	Eab7_2279	Flagellin domain protein	-	
	Eab7_2280	Hypothetical protein	-	
	Eab7_2281	Hypothetical protein	-	
	Eab7_2282	Diguanylate cyclase	-	
	Eab7_2283	Hypothetical protein	-	
	Eab7_2284	Hypothetical protein	-	
	Eab7_2285	Flagellar protein FliS	*fliS*	
	Eab7_2286	Flagellar hook-associated 2 domain protein	*fliD*	
	Eab7_2287	Flagellar protein	-	
	Eab7_2288	LAGLIDADG DNA endonuclease	-	
	Eab7_2289	Flagellin domain protein	-	
	Eab7_2290	Methyl-accepting chemotaxis sensory transducer	-	
	Eab7_2291	Extracellular solute-binding protein family 5	-	
	Eab7_2292	Hypothetical protein	-	
	Eab7_2293	Hypothetical protein		
	Eab7_2294	GCN5-related N-acetyltransferase	-	
	Eab7_2295	Hypothetical protein	-	
	Eab7_2296	Hypothetical protein	-	
	Eab7_2297	Integral membrane sensor signal transduction histidine kinase	-	
	Eab7_2298	Two component transcriptional regulator, winged helix family	-	
	Eab7_2299	Hypothetical protein	-	

	Eab7_2300	Diguanylate cyclase/phosphodiesterase with PAS/PAC sensor(S)	-	
	Eab7_2301	Hypothetical protein	-	
	Eab7_2302	Hypothetical protein	-	
	Eab7_2303	Squalene/phytoene synthase	-	
EaPAI_2 / EaRI_3 / EaSI_1				2459471..2469289
	Eab7_2492	Hypothetical protein	-	
	Eab7_2493	Hypothetical protein	-	
	Eab7_2494	UDP-N-acetylglucosamine 2-epimerase	*wecB*	
	Eab7_2495	Uracil phosphoribosyltransferase	*upp*	
	Eab7_2496	Hypothetical protein	-	
	Eab7_2497	Hypothetical protein	-	
	Eab7_2498	Hypothetical protein	-	
	Eab7_2499	Two-component sensor histidine kinase	-	
	Eab7_2500	DNA-binding response regulator	-	
	Eab7_2501	Serine hydroxymethyltransferase	*glyA*	
EaRI_1				1977107..1985836
	Eab7_2022	Ribonuclease HIII	*rnhC*	
	Eab7_2023	UDP-N-acetylmuramoyl-L-alanyl-D-glutamate--2, 6-diaminopimelate ligase	*murE*	
	Eab7_2024	Hypothetical protein	-	
	Eab7_2025	Hypothetical protein	-	
	Eab7_2026	MarR family	-	
	Eab7_2027	Major Facilitator Superfamily	-	
	Eab7_2028	ABC transporter	-	
	Eab7_2029	Hypothetical protein		

[a] Pseudogene.

Table S2. Values of nonsynonymous (ka) and synonymous (Ks) nucleotide substitutions for each branch of the tree of Fig. S2.

Node#	Ka/Ks Branch1	Ka Branch1	Ks Branch1	Ka/Ks Branch2	Ka Branch2	Ks Branch2
**1**	0.1255	0.08568178	0.6825	0.3256	0.1139	0.3499
**2**	0.2177	0.08821894	0.4052	0.08042354	0.00128370	0.01596172
**3**	0.3501	0.00558702	0.01595635	0.2296	0.00559607	0.02437185
**4**	6.8891	0.00688914	1e-10	2.2892	0.00228923	1e-10
**5**	7.6612	0.00766117	1e-10	8.2799	0.00827992	1e-10

Table S3. List of CDSs shared between *Psychrobacter* and *Exiguobacterium*. Core genes were identified using PGAP with a coverage cutoff value of 80% and identity of 50%.

Locus_tag	Gene name	Product
Eab7_1549	-	Cold shock protein
Eab7_0912	-	ABC transporter
Eab7_1365	*adhA*	Aldehyde dehydrogenase
Eab7_1587	*opuE*	Osmoregulated proline transporter
Eab7_0059	*pabA*	Para-aminobenzoate/anthranilate synthase glutamine amidotransferase component II
Eab7_0096	*tuf*	Translation elongation factor Tu
Eab7_1026	-	Sulfate permease
Eab7_0871	*accC*	Acetyl-CoA carboxylase, biotin carboxylase
Eab7_2409	-	Nucleotide sugar dehydrogenase
Eab7_0897	*trpB*	Tryptophan synthase beta chain
Eab7_0065	*lysS*	Lysyl-tRNA synthetase
Eab7_0014	*serS*	Seryl-tRNA synthetase
Eab7_0071	*radA*	DNA repair protein RadA
Eab7_2035	*rplT*	50S ribosomal protein L20
Eab7_2205	*hisF*	Imidazole glycerol phosphate synthase subunit HisF
Eab7_1999	*rph*	Ribonuclease PH
Eab7_1070	-	Transketolase
Eab7_2244	*clpP*	ATP-dependent Clp protease proteolytic subunit
Eab7_1702	*rpsB*	30S ribosomal protein S2
Eab7_2576	*groL*	60 kDa chaperonin
Eab7_2354	*gatA*	Glutamyl-tRNA(Gln) amidotransferase subunit A
Eab7_0074	*ispF*	2-C-methyl-D-erythritol 2,4-cyclodiphosphate synthase
Eab7_1933	-	Aspartate--tRNA ligase
Eab7_1104	-	Aconitate hydratase
Eab7_1574	*dhaS*	Aldehyde dehydrogenase dhaS
Eab7_1741	*sucD*	Succinyl-CoA ligase [ADP-forming] subunit alpha
Eab7_2259	*uvrB*	UvrABC system protein B
Eab7_1850	*typA*	GTP-binding protein TypA/BipA
Eab7_1628	*panD*	Aspartate 1-decarboxylase
Eab7_0085	*rplK*	50S ribosomal protein L11
Eab7_0833	*pstB*	Phosphate import ATP-binding protein pstB
Eab7_0433	*purH*	Bifunctional purine biosynthesis protein purH
Eab7_0110	*rplE*	50S ribosomal protein L5
Eab7_1742	*sucC*	Succinyl-CoA ligase [ADP-forming] subunit beta
Eab7_0009	*guaB*	Inosine-5`-monophosphate dehydrogenase

Eab7_0121	*infA*	Translation initiation factor IF-1
Eab7_2483	*atpD*	ATP synthase subunit beta
Eab7_0104	*rpsC*	Ribosomal protein S3
Eab7_1944	*ruvB*	Holliday junction ATP-dependent DNA helicase RuvB
Eab7_2501	*glyA*	Serine hydroxymethyltransferase
Eab7_0749	*lepA*	Elongation factor 4
Eab7_2840	*rpsR*	30S ribosomal protein S18
Eab7_0108	*rplN*	50S ribosomal protein L14
Eab7_0423	*purE*	N5-carboxyaminoimidazole ribonucleotide mutase
Eab7_1247	-	Serine/threonine transporter SstT
Eab7_0097	*rpsJ*	30S ribosomal protein S10
Eab7_1157	*thiG*	thiazole synthase
Eab7_2236	*eno*	Enolase
Eab7_0123	*rpsK*	30S ribosomal protein S11
Eab7_0115	*rpsE*	ribosomal protein S5
Eab7_2258	*uvrA*	Excinuclease ABC, A subunit
Eab7_1651	*hupA*	DNA-binding protein HU
Eab7_0122	*rpsM*	30S ribosomal protein S13
Eab7_2543	*pyrG*	CTP synthase
Eab7_0799	*glyQ*	glycyl-tRNA synthetase subunit alpha
Eab7_0094	*rpsG*	30S ribosomal protein S7
Eab7_2534	*rho*	Transcription termination factor Rho
Eab7_0130	*rplM*	50S ribosomal protein L13
Eab7_1954	*rpmA*	50S ribosomal protein L27
Eab7_0506	-	Aldehyde dehydrogenase
Eab7_0176	-	hypothetical protein
Eab7_0131	*rpsI*	30S ribosomal protein S9
Eab7_2522	*ribH*	6,7-dimethyl-8-ribityllumazine synthase
Eab7_2843	-	GTP-binding protein YchF
Eab7_1910	*fabF*	3-oxoacyl-[acyl-carrier-protein] synthase 2
Eab7_1925	*mnmA*	tRNA-specific 2-thiouridylase MnmA
Eab7_1684	*rpsO*	30S ribosomal protein S15
Eab7_0098	*rplC*	50S ribosomal protein L3
Eab7_2131	*metK*	S-adenosylmethionine synthase
Eab7_0102	*rpsS*	30S ribosomal protein S19
Eab7_0754	*dnaK*	Chaperone protein DnaK

Eab7_0448	*clpB*	Chaperone protein ClpB
Eab7_0105	*rplP*	50S ribosomal protein L16
Eab7_1595	*fumC*	Fumarate hydratase class II
Eab7_2809	-	Peroxiredoxin
Eab7_1334	*arsB*	Arsenical pump membrane protein
Eab7_0998	-	recA protein
Eab7_0101	*rplB*	50S ribosomal protein L2
Eab7_0111	*rpsN*	30S ribosomal protein S14
Eab7_0095	*fusA*	Elongation factor G
Eab7_1573	-	hypothetical protein
Eab7_1942	*tgt*	Queuine tRNA-ribosyltransferase
Eab7_2507	*prfA*	Peptide chain release factor 1
Eab7_0088	*rplL*	50S ribosomal protein L7/L12
Eab7_2485	*atpA*	ATP synthase subunit alpha
Eab7_0107	*rpsQ*	30S ribosomal protein S17
Eab7_2577	*groS*	10 kDa chaperonin
Eab7_2700	*ung*	Uracil-DNA glycosylase
Eab7_1981	*hemL2*	Glutamate-1-semialdehyde 2,1-aminomutase 2
Eab7_1962	*mreB*	Rod shape-determining protein MreB
Eab7_1993	*clpX*	ATP-dependent Clp protease ATP-binding subunit ClpX
Eab7_0103	*rplV*	50S ribosomal protein L22
